# Supplementary material for: Evidence and Metabolic Implications for a New Non-Canonical Role of Cu-Zn Superoxide Dismutase
Source: Int J Mol Sci. 2023 Feb 6;24(4):3230. doi: 10.3390/ijms24043230 (PMC9966940; doi:10.3390/ijms24043230)
Supplement: Supplementary file 1 [file ijms-24-03230-s001.zip › ijms-2139891-supplementary.pdf]

Supplementary Experimental Methods and Materials

S1. Impact of Oxidative Stress on Protein–Protein Interactions

HEK293T cells were seeded and cultured with the same process for standard PCA described above. On transfection day, the cells were cotransfected with YWHAE-F2N and WT-SOD1-F1N (for oxidant generators treatment) or SOD1-H46R-F1N or SOD1-D124N-F1N (for antioxidant treatment) as representative PPIs. At 48 h post transfection, cells were treated with diquat, TBHP, NAC, CuDIPs, and ebselen to a final concentration of 50 μM or the same hourly for 5 h using Tecan M1000 spectrophotometer at excitation = 512 nm and emission = 529 nm. Relative fold change was normalized by the 0 h initial point. The time and dosage selection of treatments were based on preliminary results.

S2. Protein Complex Structure Prediction

The protein complex is predicted by AlphaFold2 (Colab platform) using MMseqs2 [1,2]. The monomer and dimer complexes are based on the human protein sequences of SOD1, YWHAE, and YWHAZ from Uniprot. The settings were maintained as default, but the user amber was selected to generate a relaxed model, which is more accurate. The highest-ranking 3D coordinate structure was visualized in Pymol (The PyMOL Molecular Graphics System, Version 2.0 Schrödinger, LLC.). The per-residue confidence metric (pLDDT) is used to estimate the prediction confidence of each residue (pLDDT > 90 indicates very high confidence).

1. Evans, R.; O'Neill, M.; Pritzel, A.; Antropova, N.; Senior, A.; Green, T.; Žídek, A.; Bates, R.; Blackwell, S.; Yim, J.; et al. *Protein complex prediction with AlphaFold-Multimer*; Cold Spring Harbor Laboratory: Cold Spring Harbor, NY, USA, 2021.
2. Jumper, J.; Evans, R.; Pritzel, A.; Green, T.; Figurnov, M.; Ronneberger, O.; Tunyasuvunakool, K.; Bates, R.; Žídek, A.; Potapenko, A.; et al. Highly accurate protein structure prediction with AlphaFold. *Nature* **2021**, *596*, 583–589.

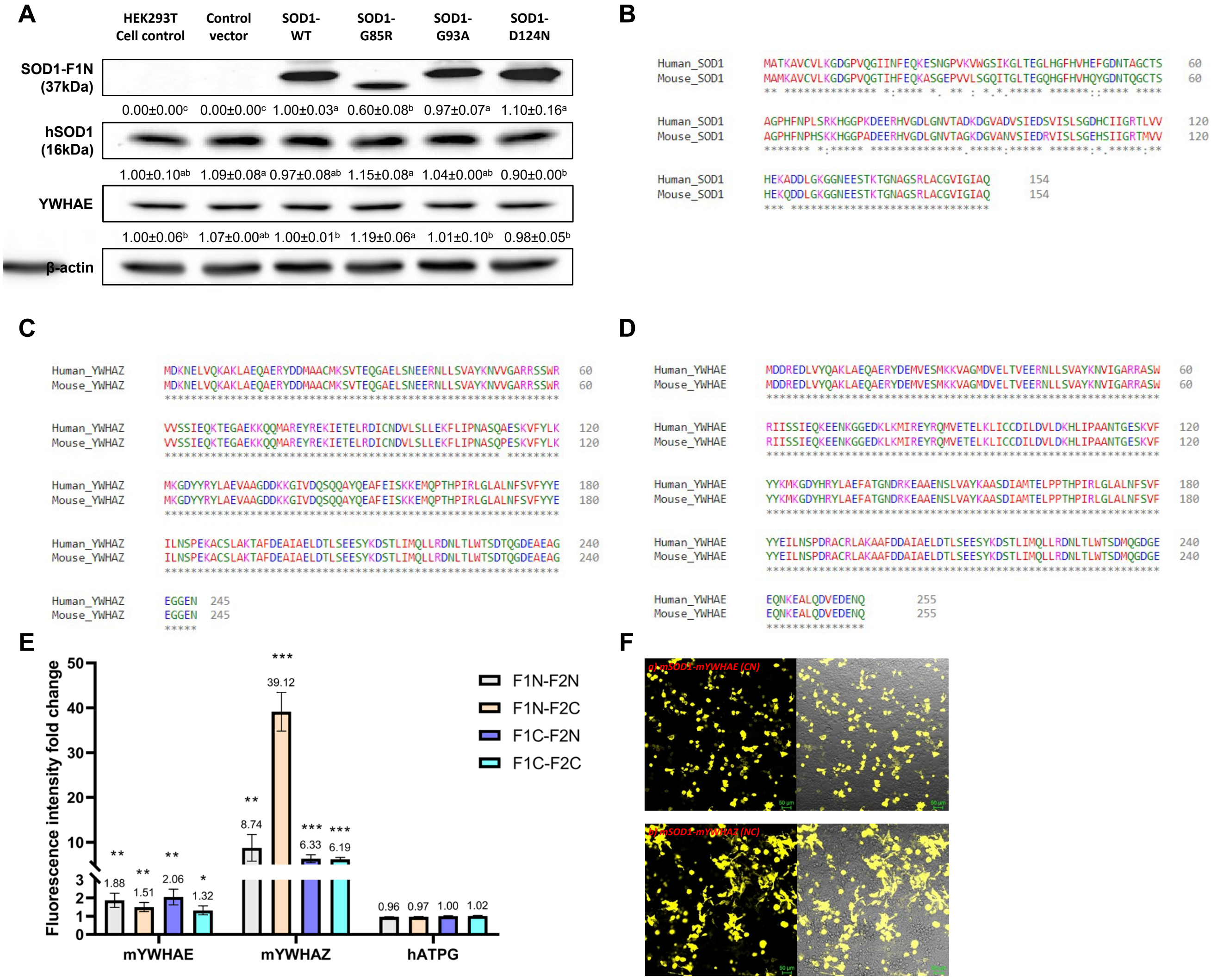

Figure S1. Additional evidence for protein–protein interactions between SOD1 and YWHAZ or YWHAE by PCA. (A) YWHAE protein expression levels in the HEK293T cells transfected with SOD1-F1N vectors or mutant SOD1-F1N (G85R, G93A, D124N). Values below individual bands are means of the relative density of two independent replicates. (B) Human and mouse SOD1 protein sequences alignment by Clustal Omega with 84% identity. (C) Human and mouse YWHAZ protein sequences alignment by Clustal Omega with 99% identity. (D) Human and mouse YWHAE protein sequences alignment by Clustal Omega with 100% identity. (E) Relative binding intensity between mSOD1 and mYWHAE or mYWHAZ at different orientations. The fluorescent intensity fold change was normalized by the control vectors pair in the same constitution(F) Representative images fluorescent microscopy of transient transfected HEK293T cells expressing PCA protein pairs. a) mSOD1-mYWHAE in F1C-F2N, b) mSOD1-mYWHAZ in F1N-F2N. . The asterisk (*) indicated the significant difference in PCA results (mSOD1-mYWHAZ or mSOD1-mYWHAE) compared with hATPG in the same orientation by Student's *t*-test. *: $p < 0.05$; **: $p < 0.01$; and ***: $p < 0.001$. n = 3–5.

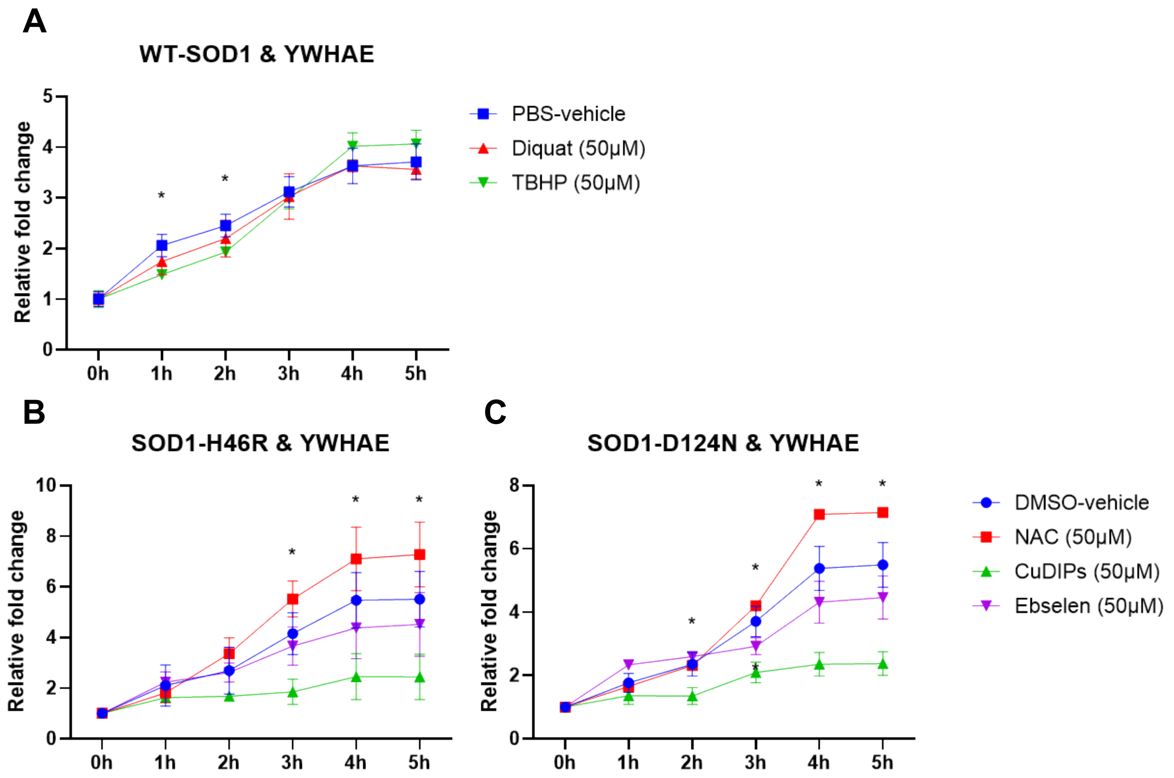

Figure S2. Impacts of redox status on the protein–protein interaction between SOD1 and YWHAE. The upturn in the curves reflected the formation of new interactions after two proteins were expressed so that the fluorescence intensity kept increasing. (A) Relative fold change fluorescence of interaction between wild-type SOD1 and YWHAE in HEK293T cells treated with PBS, 50 μM diquat, or 50 μM TBHP in 5 h. (B) Relative fold change fluorescence of interaction between mutant SOD1-H46R and YWHAE in HEK293T cells treated with vehicle solution (DMSO), 50 μM NAC, 50 μM CuDIPs, or 50 μM ebselen in 5 h. (C) Relative fold change fluorescence of interaction between mutant SOD1-D124N and YWHAE in HEK293T cells treated with vehicle solution (DMSO), 50 μM NAC, 50 μM CuDIPs, or 50 μM ebselen for 5 h. The asterisks (*) above the time points on the curves indicated significant differences between at least one treatment and control vehicle group, *: $p < 0.05$, $n = 3$.

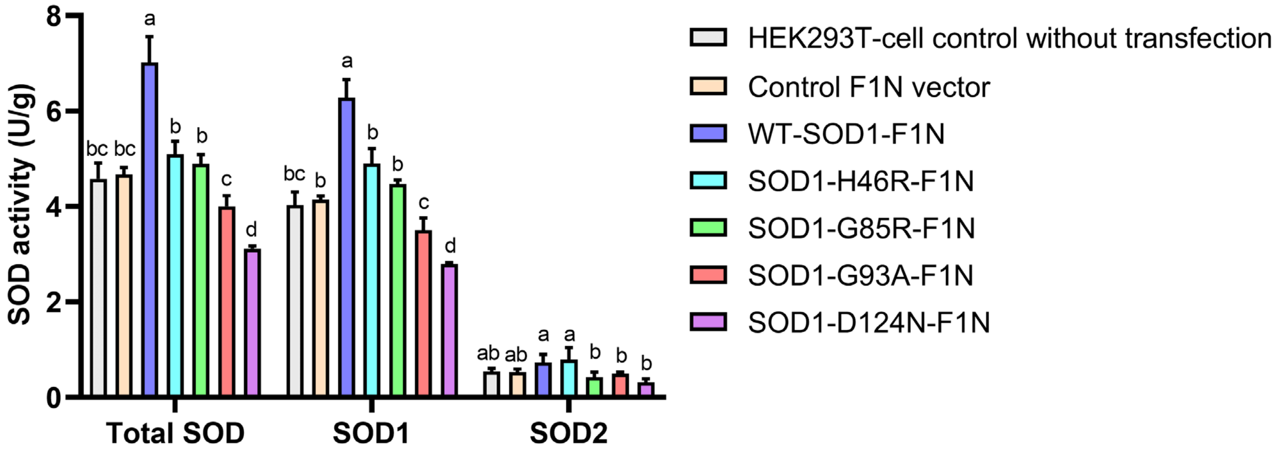

Figure S3. Intracellular total SOD, SOD1, and SOD2 activity (U/g) of HEK293T cell lysate without or with transfected with WT-SOD1-F1N or mutant SOD1-F1N (H46R, G85R, G93A, and D124N) or control vector F1N at 72 h after transfection ($n = 3$). Means within an enzyme activity without sharing a common letter differ ($p < 0.05$).

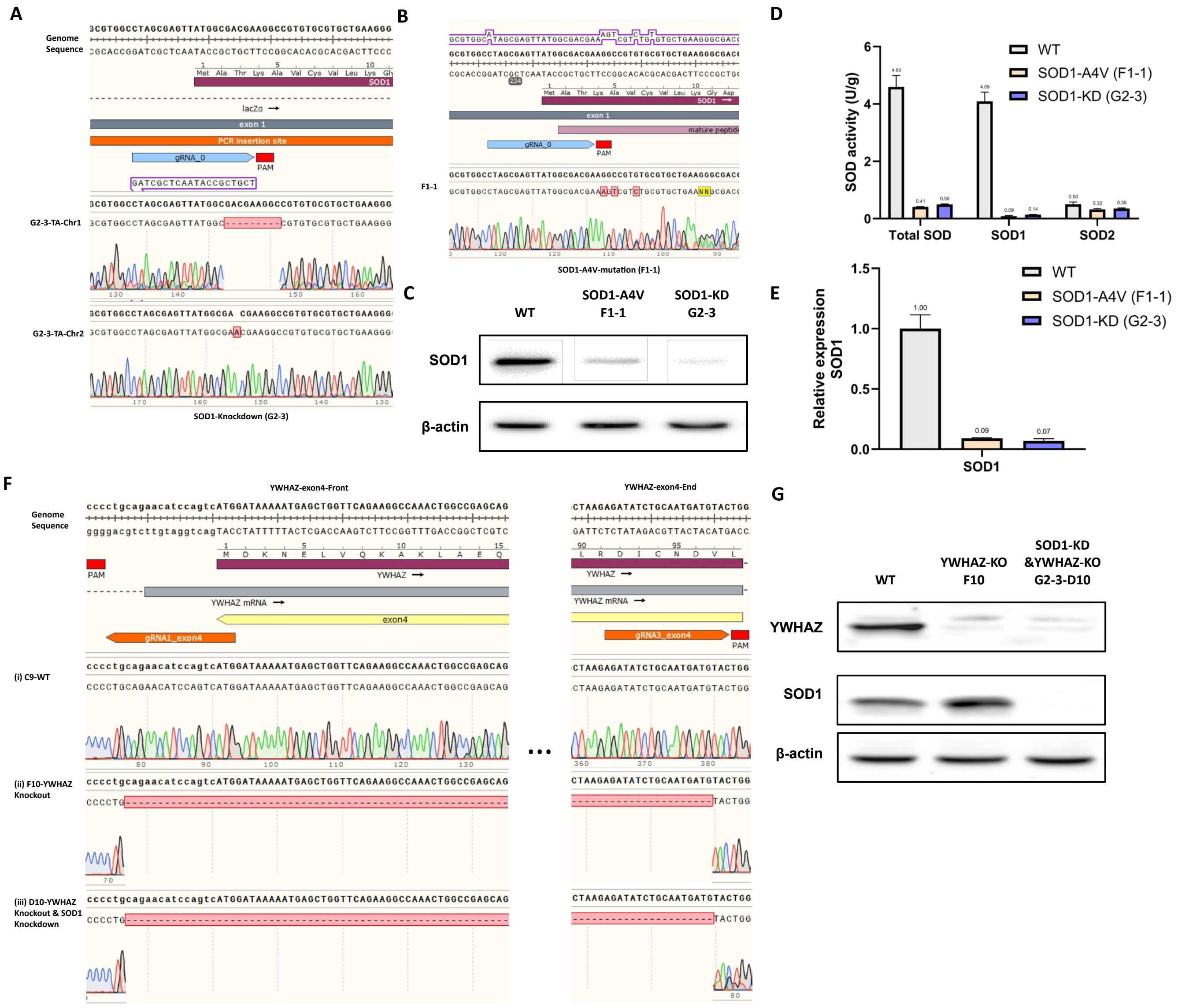

Figure S4. Evidence of successful CRISPR-edited HEK293T cells of SOD1-A4V, SOD1-KD, YWHAZ-KO, and SOD1-KD&YWHAZ-KO. (A) The *SOD1* exon1 genome Sanger sequence results in heterozygous SOD1 knockdown (SOD1-KD) HEK293T cells (ID: G2-3) with the first chromosome missing three amino acids and the second chromosome frameshift mutation after TA cloning ligation for both chromosomes. (B) The *SOD1* exon1 genome Sanger sequence results in homozygous SOD1-A4V mutation HEK293T cells (ID: F1-1). (C) Representative images of Western blot analysis of SOD1 in CRISPR-edited HEK293T cells: SOD1 protein levels in WT (Wild-type), F1-1 (SOD1-A4V), and G2-3 (SOD1 KD). (D) SOD1 family activity levels of CRISPR-edited cells. (E) *SOD1* mRNA amounts in CRISPR-edited cells relative to the beta-actin level. (F) *YWHAZ* exon4 genome Sanger sequence results in YWHAZ knockout cells: (i) Wild type HEK293T cells. (ii) YWHAZ knockout HEK293T cells (ID: F10) (iii) YWHAZ knockout cells in SOD1 knockdown (G2-3) cells (ID: G2-3-D10). (G) Representative images of Western blot analysis of SOD1 and YWHAZ proteins levels in the CRISPR-edited HEK293T cells. (i) SOD1 protein in WT (Wild-type HEK293T), F1-1 (SOD1-A4V mutation HEK293T cells), and G2-3 (SOD1 knockdown HEK293T cells). (ii) YWHAZ protein in WT (Wild-type HEK293T), F10 (YWHAZ knockout HEK293T cells), and G2-3-D10 (YWHAZ knockout and SOD1 knockdown HEK293T cells).

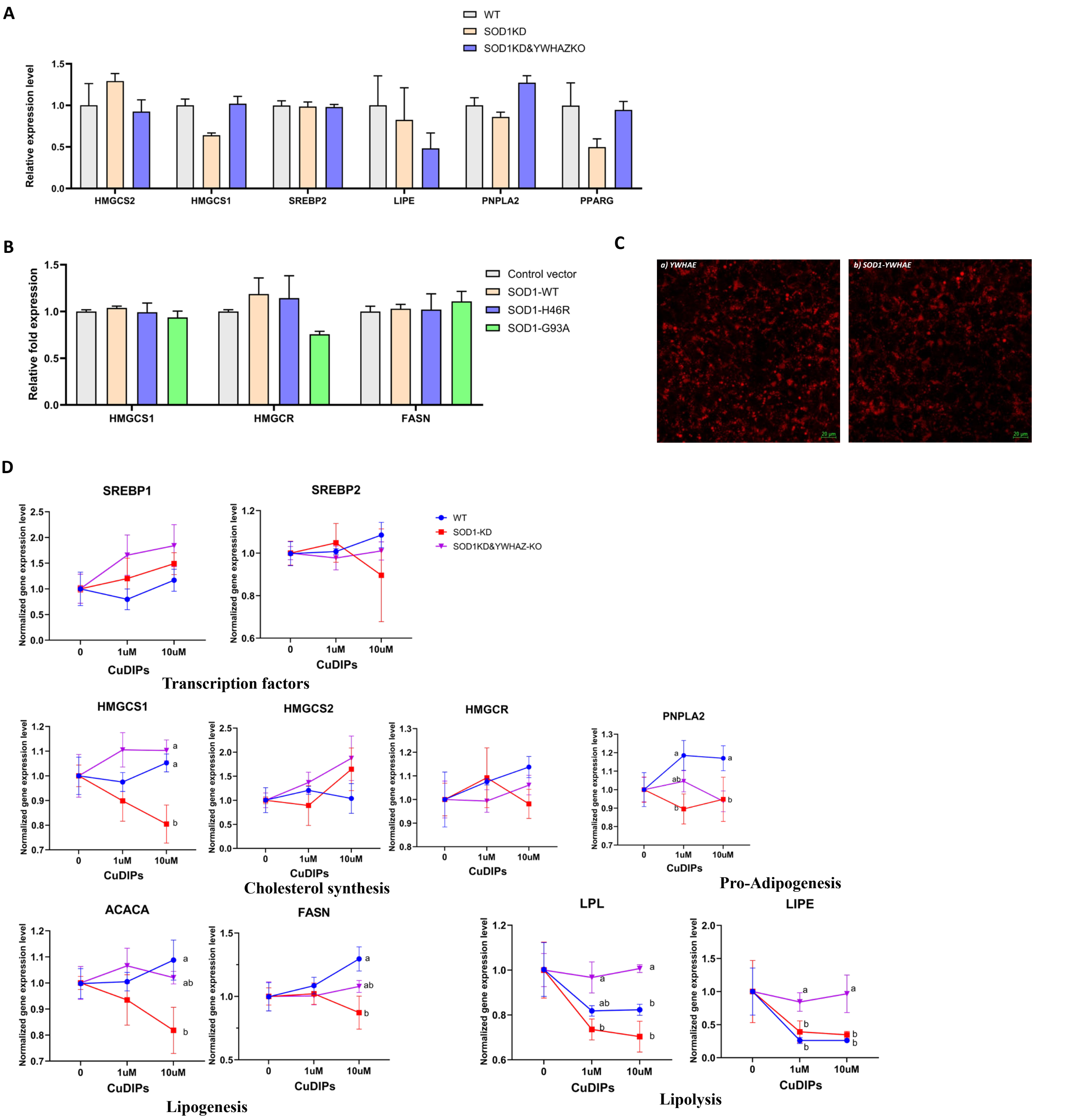

Figure S5. Additional evidence of the disrupted SOD1 and YWHAZ or YWHAE protein–protein interactions on cellular lipid metabolism. (A) Relative mRNA levels of lipid metabolism genes in WT, SOD1KD, and SOD1KD&YWHAZKO HEK293T cells ($n = 4$). (B) Relative mRNA levels of lipid metabolism genes in HepG2 cells transfected with control vector F1N, WT-SOD1, or SOD1 mutants (H46R and G93A) ($n = 4$). (C) Fluorescent images of Nile-red stained HepG2 cells cotransfected with WT-SOD1-F1N and YWHAE-F2N or YWHAE-F2N and F1N control vector. Annotation: a) YWHAE: cells cotransfected with YWHAE-F2N and F1N control vector, b) SOD1-YWHAE: cells transfected with YWHAE-F2N and SOD1-F1N, Scale bar, 20 μm. The image was representative of three sets of data. (D) Relative mRNA levels of lipid metabolism genes under treatments of graded levels of CuDIPs for 48 h in WT, SOD1KD, and SOD1KD&YWHAZKO HEK293T cells ($n = 4$). Means within a given concentration of CuDIPs without sharing a common letter differ ($p < 0.05$).

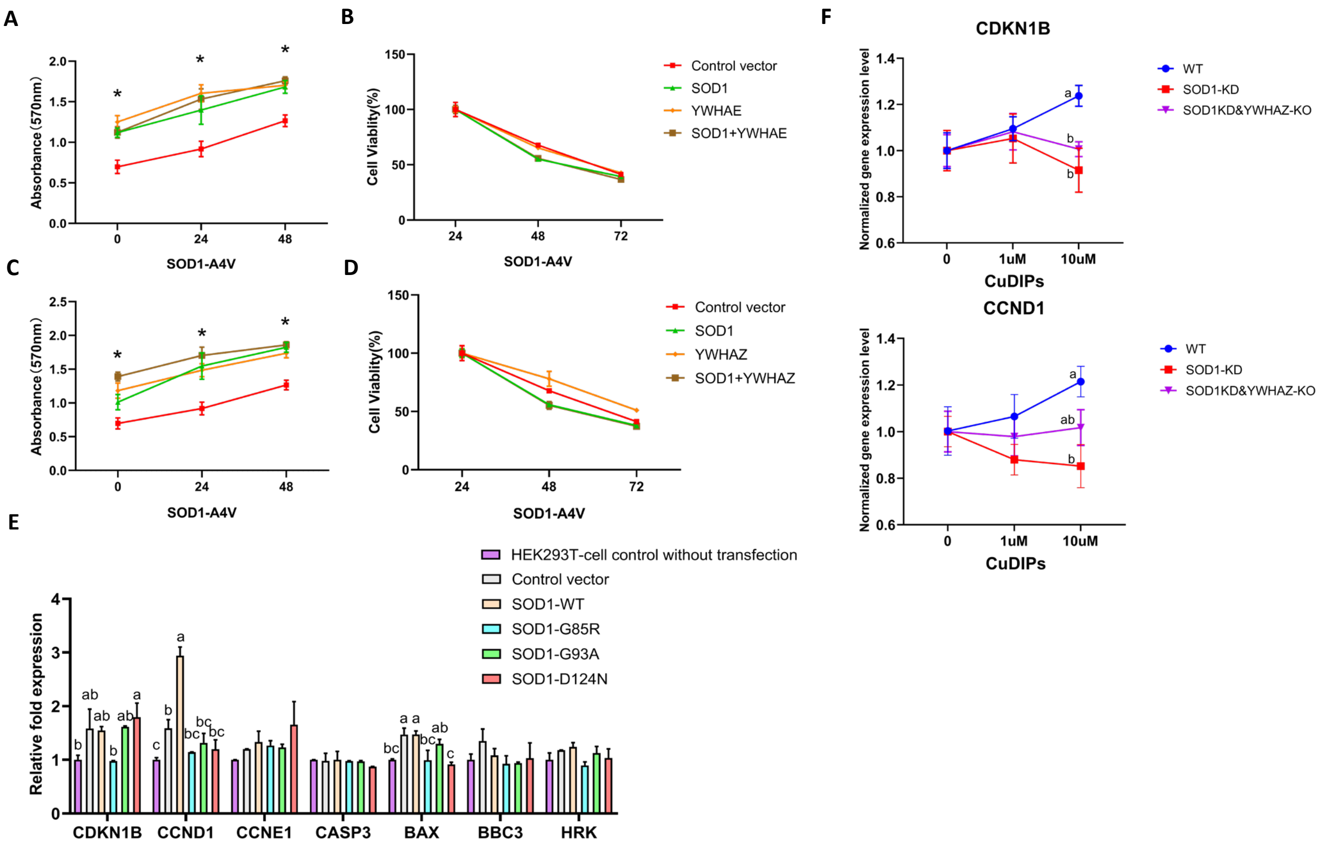

Figure S6. Additional evidence for impacts of altering protein–protein interactions between SOD1 and YWHAE or YWHAZ on cell growth and viability of HEK293T cells. (A) Proliferation curves of SOD1-A4V cells overexpressing SOD1 and YWHAE alone or in combination supplemented with FBS culture media. (B) Viability curves of SOD1-A4V cells overexpressing SOD1 and YWHAE alone or in combination after the removal of FBS in culture media. (C) Proliferation curves of SOD1-A4V cells overexpressing SOD1 and YWHAZ alone or in combination supplemented with FBS culture media. (D) Viability curves of SOD1-A4V cells overexpressing SOD1 and YWHAZ alone or in combination after the removal of FBS in culture media. (E) Relative mRNA levels of cell proliferation genes in HEK293T cells overexpressing SOD1, SOD1 mutants or control vector F1N ($n = 4$). (F) Relative *CDKN1B* and *CCND1* mRNA levels in WT, SOD1-KD, SOD1-KD&YWHAZ-KO cells under treatments of graded levels of CuDIPs for 48h ($n = 4$). In A-D, the asterisks (*) indicated that at least one treatment group was different from the negative control (F1N + F2N), $p < 0.05$, $n = 4$. Means for a given concentration of CuDIPs or gene without sharing a common letter differ ($p < 0.05$).

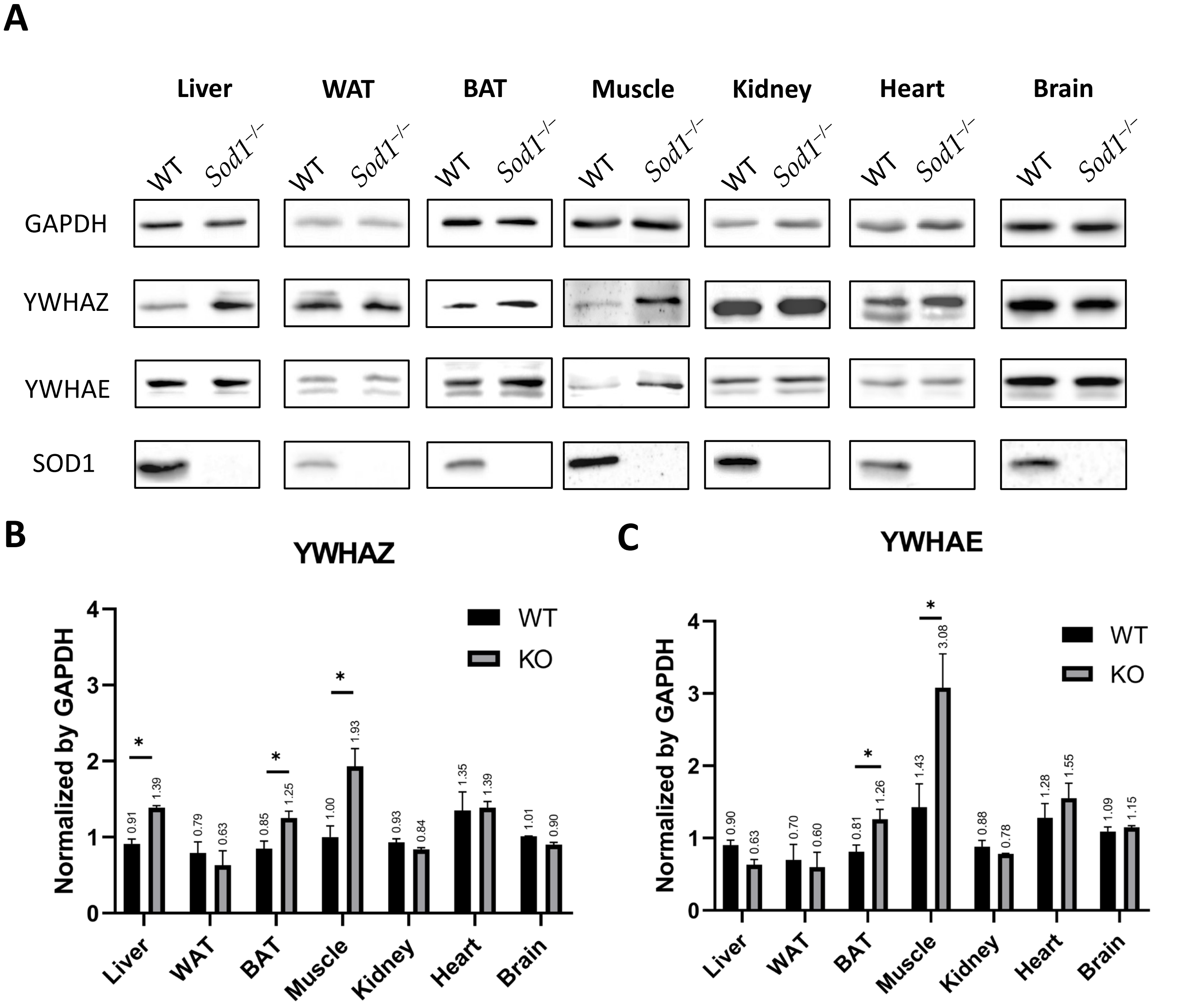

Figure S7. Protein expression levels on SOD1, YWHAE, and YWHAZ in different tissues of wild-type (WT) and *Sod1*$^{-/-}$ (KO) mice. (A) A representative image of immunoblots of YWHAZ and YWHAE protein expression levels in different tissues from wild-type and SOD1 knockout mice. The negative correlation was also reflected by increased YWHAZ protein expression in SOD1-KO mice but not consistent for YWHAE protein. (B) Quantification of the density of protein bands on YWHAZ normalized by GAPDH. (C) Quantification of the density of protein bands on YWHAE normalized by GAPDH. The asterisks indicated significant differences between KO and WT, *: $p < 0.05$, $n = 3$–5.

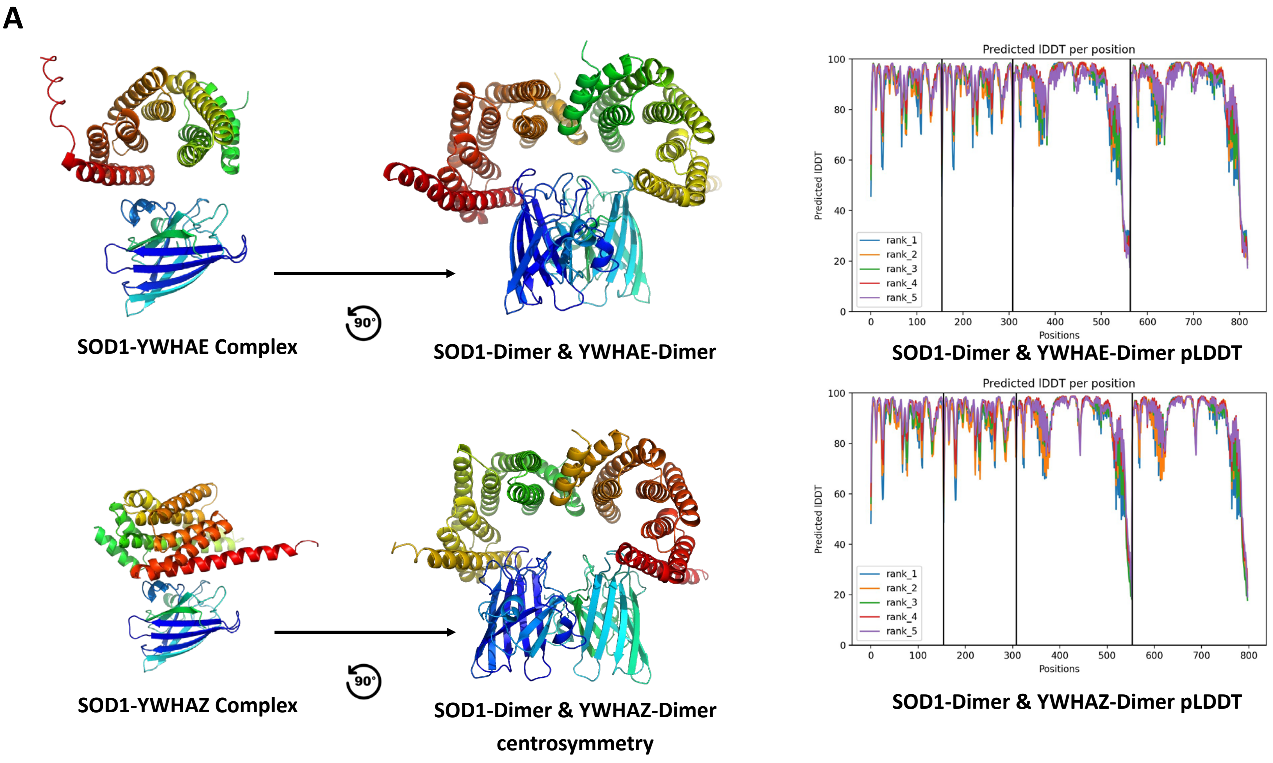

B

```
YWHAE      MDDREDLVYQAKLAEQAERYDEMVESMKKVAGMDVELTVEERNLLSVAYKNVIGARRASW      60
YWHAZ      -MDKNELVQKAKLAEQAERYDDMAACMKSVTEQGAELSNEERNLLSVAYKNVVGARRSSW      59
             *:::** :***********:*. .**.*:  ..**: *************:****:**

YWHAE      RIISSIEQKEENKGGEDKLKMIREYRQMVETELKLICCDILDVLDKHLIPAANTGESKVF      120
YWHAZ      RVVSSIEQKTE--GAEKKQQMAREYREKIETELRDICNDVLSLLEKFLIPNASQPESKVF      117
           *::****** *  *.*.* :* ****: :****: ** *:*.:*:*.*** *.  *****

YWHAE      YYKMKGDYHRYLAEFATGNDRKEAAENSLVAYKAASDIAMTELPPTHPIRLGLALNFSVF      180
YWHAZ      YLKMKGDYYRYLAEVAAGDDKKGIVDQSQQAYQEAFEISKKEMQPTHPIRLGLALNFSVF      177
           * ******:*****.*:*:*:*  .::*  **: * :*: .*: ****************

YWHAE      YYEILNSPDRACRLAKAAFDDAIAELDTLSEESYKDSTLIMQLLRDNLTLWTSDMQGDGE      240
YWHAZ      YYEILNSPEKACSLAKTAFDEAIAELDTLSEESYKDSTLIMQLLRDNLTLWTSDTQGDEA      237
           ********::** ***:***:********************************* ***

YWHAE      EQNKEALQDVEDENQ      255
YWHAZ      EAGEGGEN-------      245
           * .: . :
```

Figure S8. Protein complex structure prediction and sequence alignment. (A) Monomer and dimer complex output predicted by AlphaFold2. The pLDDT figures indicated confidence in each amino acid position. (B) YHWAE and YWHAZ protein sequence alignment by Clustal Omega with 69% identity and 81% similarity.

Table S1. The list of plasmids used in this study

Plasmids	Description	Host	Source
PCA vectors			
F1N	Integrative pcDNA3.1 vector with YFP 1st fragment in N terminal	ccdB Survival $T1^R$	Dr. Yu lab
F1C	Integrative pcDNA3.1 vector with YFP 1st fragment in C terminal	ccdB Survival $T1^R$	Dr. Yu lab
F2N	Integrative pcDNA3.1 vector with YFP 2nd fragment in N terminal	ccdB Survival $T1^R$	Dr. Yu lab
F2C	Integrative pcDNA3.1 vector with YFP 2nd fragment in C terminal	ccdB Survival $T1^R$	Dr. Yu lab
SOD1-F1N or WT-SOD1-F1N	Integrative F1N vector with Homo sapiens *SOD1* gene	DH5α $T1^R$	This study
SOD1-F1C or WT-SOD1-F1C	Integrative F1C vector with Homo sapiens *SOD1* gene	DH5α $T1^R$	This study
mSOD1-F1N	Integrative F1N vector with Mus musculus *Sod1* gene	DH5α $T1^R$	This study
mSOD1-F1C	Integrative F1C vector with Mus musculus *Sod1* gene	DH5α $T1^R$	This study
YWHAE-F2N	Integrative F2N vector with Homo sapiens *YWHAE* gene	DH5α $T1^R$	This study
YWHAE-F2C	Integrative F2C vector with Homo sapiens *YWHAE* gene	DH5α $T1^R$	This study
mYWHAE-F2N	Integrative F2N vector with Homo sapiens *Ywhae* gene	DH5α $T1^R$	This study
mYWHAE-F2C	Integrative F2C vector with Homo sapiens *Ywhae* gene	DH5α $T1^R$	This study
YWHAZ-F2N	Integrative F2N vector with Homo sapiens *YWHAZ* gene	DH5α $T1^R$	This study
YWHAZ-F2C	Integrative F2C vector with Homo sapiens *YWHAZ* gene	DH5α $T1^R$	This study
mYWHAZ-F2N	Integrative F2N vector with Mus musculus *Ywhaz* gene	DH5α $T1^R$	This study
mYWHAZ-F2C	Integrative F2C vector with Mus musculus *Ywhaz* gene	DH5α $T1^R$	This study
ATPG-F2N	Integrative F2N vector with Homo sapiens *ATPG* gene	DH5α $T1^R$	This study
ATPG-F2C	Integrative F2C vector with Homo sapiens *ATPG* gene	DH5α $T1^R$	This study
SOD1 mutants			
SOD1-H46R-F1N	SOD1 gene has H46R: CAT → CGT mutation in SOD1-F1N vector	DH5α $T1^R$	This study
SOD1-H46R-F1C	SOD1 gene has H46R: CAT → CGT mutation in SOD1-F1C vector	DH5α $T1^R$	This study
SOD1-G85R-F1N	SOD1 gene has G85R: GGC → CGC mutation in SOD1-F1N vector	DH5α $T1^R$	This study
SOD1-G85R-F1C	SOD1 gene has G85R: GGC → CGC mutation in SOD1-F1C vector	DH5α $T1^R$	This study
SOD1-G93A-F1N	SOD1 gene has G93A: GGT → GCT mutation in SOD1-F1N vector	DH5α $T1^R$	This study
SOD1-G93A-F1C	SOD1 gene has G93A: GGT → GCT mutation in SOD1-F1C vector	DH5α $T1^R$	This study
SOD1-D124N-F1N	SOD1 gene has D124N: GAT → AAT mutation in SOD1-F1N vector	DH5α $T1^R$	This study
SOD1-D124N-F1C	SOD1 gene has D124N: GAT → AAT mutation in SOD1-F1C vector	DH5α $T1^R$	This study
Protein Expression			
pX459-*SOD1*	Integrative pX459 vector with gRNA targeting *SOD1* exon1 region 5′- CTAGCGAGTTATGGCGACGA-3′	DH5α $T1^R$	This study
pDG459-*YWHAZ*	Integrative pDG459 vector with gRNA targeting *YWHAZ* exon4 5′-CATGACTGGATGTTCTGCAG-3′ and 5′-AGATATCTG-CAATGATGTAC-3′	DH5α $T1^R$	This study
ssODN-SOD1-A4V	5'- TTCCGTTGCAGTCCTCGGAACCAGGACCTCGGCGTGGCATAGCGA GTTATGGCGACGAAAGTCGTCTGTGTGCTGAAGGGCGACGGCCCAG TGCAGGGCATCATCAATTTCGAGCAGAAGGCA -3′		
SOD1-pPICZα1-His	Integrative pPICZα1 vector with *SOD1* gene for protein expression	X33	This study
YWHAE-pPICZα1-His	Integrative pPICZα1 vector with *YWHAE* gene for protein expression	X33	This study
YWHAZ-pPICZα1-His	Integrative pPICZα1 vector with *YWHAZ* gene for protein expression	X33	This study
pGEX6P1-GST-YWHAE	Integrative pGEX6P1 vector with *YWHAE* gene for protein expression	BL21(DE3)	This study
pGEX6P1-GST-YWHAZ	Integrative pGEX6P1 vector with *YWHAZ* gene for protein expression	BL21(DE3)	This study

Table S2. The list of primers used in this study

Primers	Description (5'→3')	
Mutagenesis		
SOD1_A4V.F	GCGACGAAGGTCGTGTGCGTG	
SOD1_A4V.R	CATGCCAACTTTTTTGTACAAACTTGTGATATCAG	
SOD1_H46R.F	GCATGGATTCCGTGTTCATGAGTTTG	
SOD1_H46R.R	AGGCCTTCAGTCAGTCCT	
SOD1_G85R.F	TGGAGACTTGCGCAATGTGAC	
SOD1_G85R.R	ACATGCCTCTCTTCATCC	
SOD1_G93A.F	GACAAAGATGCTGTGGCCGATG	
SOD1_G93A.R	AGCAGTCACATTGCCCAA	
SOD1_D124N.F	TGAAAAAGCAAATGACTTGGGCAAAG	
SOD1_D124N.R	TGGACCACCAGTGTGCGG	
CRISPR-Cas9 genome editing		
SOD1_gRNA.F	CACCGCTAGCGAGTTATGGCGACGA	
SOD1_gRNA.R	AAACTCGTCGCCATAACTCGCTAGC	
YWHAZ_gRNA1.F	CACCGCATGACTGGATGTTCTGCAG	
YWHAZ_gRNA1.R	AAACCTGCAGAACATCCAGTCATGC	
YWHAZ_gRNA2.F	ACCGAGATATCTGCAATGATGTACGT	
YWHAZ_gRNA2.R	TAAAACGTACATCATTGCAGATATCT	
Sequencing primers		
SOD1_exon1_seq.F	CGGAGGTCTGGCCTATAAAGTA	
SOD1_exon1_seq.R	GCTCCTAGCAAAGGTGCG	
YWHAZ_exon4_seq.F	TTTCACAGGCAGTTAGGTTTCA	
YWHAZ_exon4_seq.R	TGGAACACACAATGTTTAGAAGG	
qPCR primers (*Homo Sapiens*)		
SREBP1 (SREBP1a)	GAGGCCAGGGCAGGACA	CGCCGACTTCACCTTCG
SREBP2	GAGAAAGGCGGACAACCCAT	CGCCAGACTTGTGCATCTTG
HMGCS1	CAGTGGCAGAAAGAGGGAAAT	TTCAGCAACATCCGAGCTAGA
HMGCS2	TTGGCCTCGGAGTACCCAAT	TCGCTGCCAGCTTGCTT
HMGCR	GCCCTCAGTTCCAACTCACA	CAAGCTGACGTACCCCTGAC
ACACA	CCGAGAAAGCAGGGGATCTG	TGTTCTCCAACTCCTTCCGC
FASN	GTCTTGAACTCCTTGGCGGA	AGGAAGATAGCCATGCCGAG
LPL	CAGACAAGCAAACAACGCCA	TGAGCCTTGAGGCTGTATCC
LIPE	CAGACAAGCAAACAACGCCA	TGAGCCTTGAGGCTGTATCC
PNPLA2	AGGCTGGTGCCAAGTTCATT	GCAGACATTGGCCTGGATGA
PPARG	GGTGACCAGAAGCCTGCATT	TGTCAACCATGGTCATTTCGTT
FABP4	ACAGGAAAGTCAAGAGCACCAT	AACTCTCGTGGAAGTGACGC
TP53	ACCTATGGAAACTACTTCCTGAAAGCAGGGGAGTACGTGCAA	
DTL	AGTCCCAGTTCCTCCTTTTGG	TTCCAGTGAGCCATCCATTCTTT
CDKN1B	GCAAGTACGAGTGGCAAGAG	TTGTTTTGAGTAGAA-<br>GAATCGTCGG
CCND1	CACCTGGATGCTGGAGGTCTG	CAGGGGGATGGTCTCCTTCA
CCNE1	CAGGGAGCGGGATGCGA	TGGTGCAATAATCCGAGGCT
CASP3	TGCTATTGTGAGGCGGTTGT	CCGAGATGTCATTCCAGTGCT
BAX	TGGAGCTGCAGAGGATGATTG	ACAGGGACATCAGTCGCTTC
BBC3	GGATGAAATTTGGCATGGGGT	CCCTGGGGCCACAAATC
HRK	GCAGGCGGAACTTGTAGGAA	CAGGGTTTTCACCAACCTGT

Table S3. The list of antibodies information used in this study

Protein	Antibody Manufacturer/Catalog
Primary antibody	
SOD1	Santa Cruz Biotechnology, sc-17767
YWHAE	AB Clonal, A1058
YWHAZ	Proteintech, 14881-1-AP
Beta-Actin	Bio Vision, 3917-30T
GAPDH	Proteintech, 60004-1-Ig
Beta-Tubulin	Proteintech, 66240-1-Ig
6*His	Proteintech, 66005-1-Ig
Secondary antibody	
Goat Anti-Rabbit IgG (H+L)-HRP conjugate	BIO-RAD, 170-6515
Goat Anti-Mouse IgG (H+L)-HRP conjugate	BIO-RAD, 170-6516
VeriBlot for IP Detection Reagent (HRP)	Abcam, ab131366
